# Beijing Pinggu Childhood Eye Study: The Baseline Refractive Characteristics in 6- to 12-Year-Old Chinese Primary School Students

**DOI:** 10.3389/fpubh.2022.890261

**Published:** 2022-05-27

**Authors:** Yan Li, Yi Xing, Chunlin Jia, Jiahui Ma, Xuewei Li, Jingwei Zhou, Chenxu Zhao, Haijun Zhang, Lu Wang, Weihong Wang, Jia Qu, Mingwei Zhao, Kai Wang, Xin Guo

**Affiliations:** ^1^Beijing Key Laboratory of Diagnosis and Therapy of Retinal and Choroid Diseases, Department of Ophthalmology, Peking University People's Hospital, Beijing, China; ^2^School of Public Health, Institute of Child and Adolescent, Peking University, Beijing, China; ^3^Pinggu District Primary and Secondary School Health Care Institute, Beijing, China; ^4^Children and Adolescent Health, Beijing Center for Disease Prevention and Control, Beijing, China; ^5^Pinggu Center for Disease Prevention and Control, Beijing, China; ^6^School of Optometry and Ophthalmology and Eye Hospital, Wenzhou Medical University, Wenzhou, China

**Keywords:** primary school, refractive error, myopia, cycloplegia, ocular biometry

## Abstract

**Purpose:**

To report the design and baseline data of a 3-year cohort study in Beijing Pinggu District primary school students in China after COVID-19.

**Methods:**

Noncycloplegic and cycloplegic spherical equivalent refraction (SER) were measured, ocular biometry, including the axial length (AL), anterior chamber depth (ACD) and corneal power (CP), were collected before cycloplegia. Corneal radius (CR), AL-to-CR ratio, and lens power (LP) were calculated.

**Results:**

Among the 4,806 (89.1%) eligible students (51.5% male), the prevalence of emmetropia, myopia, mild hyperopia, and mild-to-high hyperopia was 12.8, 30.8, 53.0, and 3.3% after cycloplegia, respectively. Myopia increased from 2.5% in 6- to 71.6% in 12-year-old students, with 9- and 10-year-olds showing the most prominent increases. The median of cycloplegic SER was 0.50 (IQR = 1.63), and the noncycloplegic SER was −0.38 D (IQR = 1.50), which is more negative than the cycloplegic refraction. The mean AL increased with age, from 22.46 ± 0.70 mm to 24.26 ± 1.07 mm. The ACD increased from 3.38 ± 0.28 mm to 3.70 ± 0.30 mm, and the AL-to-CR ratio increased from 2.91 ± 0.08 to 3.12 ± 0.13 between 6- and 12-year-old students. AL, CR and LP explained the SER variance with *R*^2^ of 86.4% after adjusting the age and gender.

**Conclusions and Relevance:**

The myopia prevalence since emergence of COVID-19 rapidly increased from 6- to 12-year primary school Chinese children, especially after 7 years of age. The non-cycloplegia SER overestimated the prevalence of myopia, and the cycloplegic SER is a more accurate and reliable method to assess the prevalence of refractive status.

## Introduction

Emmetropization is the result of both passive and active processes, including a proportional enlargement of the eye and optically guided feedback of image focus in childhood (1, 2). The trend toward lessening hyperopia or increasing myopia during childhood continues with a rising prevalence of myopia each passing year, especially in East Asian populations such as in China, Japan, and Singapore (3). This increasing magnitude of myopia increases the risk of the development of irreversible disorders that can be sight threatening (4).

In China, the prevalence of myopia in school children increases annually. In a country-based survey that was conducted in 2018, the overall prevalence of myopia in 6–18-year-olds was 53.6%, and it was approximately 81.0% in senior high school students. It is estimated that the cumulative incidence of myopia would increase to 61.8% by 2030 for school-age children without effective interventions (5). Recently, data from the Myopia Epidemiology and Intervention Study in 2020 confirmed the increasing trend of myopia in China, and indicates that the overall myopia prevalence under non-cycloplegia condition is 59.35% and that the high myopia prevalence is 4.99% (6).

Evaluation of the prospective cross-sectional study refraction status of students under cycloplegia and non-cycloplegia, together with ocular biometry, will enhance our understanding of the myopia epidemic, especially in China. However, previous studies reported the individual ocular parameters separately (7–9), evaluated them with a non-cycloplegia situation (10), or reported narrow age results (1). In addition, several studies were conducted approximately 10 years ago (11, 12).

The present Beijing Pinggu Childhood Eye Study (BPCE) is a cohort study which was initiated in 2021. BPCE study aims to further understand the development of refractive status of selected students, the refractive characteristics and biometry changes during the current condition in China, In the present manuscript, we aim to report the baseline characteristics for the 3-year study, and further information will be reviewed yearly.and the influencing factors of myopia diagnosis with or without cycloplegia.

## Methods

### Study Population

The BPCE study is a school-based cohort study for three consecutive years. Students aged 6–12 years from 8 primary schools in Pinggu District of Beijing, China were prospectively selected to evaluate the visual acuity, refractive status and ocular. In the present manuscript, we aim to report the baseline characteristics for the 3-year study, and further information will be reviewed yearly.

The present project has been initiated by the Beijing Centers for Disease Control and Prevention (BJCDC) as a public health program designed to establish refraction development data in Beijing city. The investigation will be annually conducted in primary schools from January 2021 to December 2023. The present project conforms to the tenets of the Declaration of Helsinki. Ethics approval was obtained from the institutional review board of the Peking University People's Hospital (2021PHB322-001). After the study purposes and procedures were explained to the parents or legal guardians in detail during a school seminar, written informed consent was obtained.

The schools were selected due to 85% students in these schools promised to follow up the study in the 3 years. Once the schools were selected, 2–3 classes in every grade were randomly selected and all students in the classes were recruited to ensure at least 80 students in every grade in the study. Of them, 4,942 (91.7%) signed informed consent forms, and 4,806 (89.1%) children successfully completed all biometry examinations and cycloplegic refraction examinations and were eligible for analysis.

### Measurements

The distance VA was estimated using Early Treatment Diabetic Retinopathy Study Tumbling E charts (Precision Vision, La Salle, IL, USA). Ocular biometry was measured before pupil dilation with non-contact partial-coherence laser interferometry (IOLMaster 500; Carl Zeiss Meditec, Oberkochen, Germany) (13).

The non-cycloplegia autorefraction was measured first. Then, cycloplegia was induced with 2 drops of 1% cyclopentolate, administered 5 min apart, with a third drop administered 20 min later. The cycloplegia and pupil dilation were evaluated after an additional 15 min. Refraction was performed with a desktop autorefractor (KR8800; Topcon Corp., Tokyo, Japan) in participants with their pupils dilated to at least 6 mm, and when their pupillary light reflex was absent. The data on the spherical and cylindrical power and axis were extracted from the device.

The trained and certified examiners (three optometrists, two public health physicians, and one ophthalmologist) were from Peking University People's Hospital. The examinations were performed according to standard measurement procedures. All the participants with uncorrected visual acuity (VA >0.0 logMAR) received recommendations to be referred to eye care practitioners for further detailed ophthalmic examinations and evaluations.

### Definitions

Uncorrected VA higher than 0.0 logMAR was defined as referral vision. The spherical equivalent refraction (SER) was calculated as the sphere power (D) + 0.5 × cylinder power (D). Myopia was defined as an SER of −0.50 D or less, emmetropia was defined as −0.50 D < SER < +0.50 D, mild hyperopia was defined as +0.50 D ≤ SER < +2.00 D, and mild-to-high hyperopia was defined as an SER of ≥+2.00D.

Because there was high correlation between the right and left eyes of the same individual, only the biometry parameters from right eyes were presented. The corneal radius (CR) was calculated as the mean of the flattest and steepest corneal power (CP) and was converted using the formula: corneal power (D) = 0.3375/CR (mm) × 1,000; Guo et al. (13) the AL-to-CR ratio (AL/CR) was computed as the AL in millimeters divided by the CR in millimeters. The lens power was calculated based on the modified Bennett-Rabbetts formula (14).

### Statistical Analysis

Analysis of variance (ANOVA) was used to compare the biometric measurements and Chi - square were used to compare the prevalence of refractive error across different ages. Paired samples *T*-tests were performed to explore the difference of SER pre- and post- cycloplegia. Non-parametric tests were used to compare the prevalence of refractive error between pre- and post- cycloplegia. Multiple regression analysis was performed to explore the associations between the refraction and ocular biometry. All analyses were performed using IBM SPSS Statistics 26.0 software. A *P*-value of 0.05 or less was considered statistically significant.

## Results

A total of 4,806 (89.1%) children were included in the final analysis (Supplementary Figure 1). Of these, 2,477 were boys (51.5%) and 2,329 were girls (48.5%) with no significant gender difference in each age group (χ^2^ = 3.817, ρ = 0.791).

Table 1 demonstrates the prevalence of emmetropia, myopia, mild hyperopia, and mild-to-high hyperopia was 12.8, 30.8, 53.0, and 3.3% after cycloplegia, respectively; and was 28.5, 53.1, 17.5, and 0.9% before cycloplegia. Myopia prevalence increased from 2.5% at 6 years of age to 71.6% at 12 years of age, with a speed increase of 27.6%. Remarkably, the myopic children at 9- and 10-years old increase significantly, with the increased prevalence was 17.2 and 15.2% separately compared to the prior age group. Significant difference in the refraction status between pre- and post-cycloplegia treatment was found. The myopia prevalence decreased by 22.3% compared with that before cycloplegia. With increasing age, the myopia prevalence became closer within the age groups compared with pre- and post- cycloplegia treatment (Tables 1, 2). There was no significant difference in the refraction status between girls and boys (χ^2^ = 0.133, ρ = 0.067). The prevalence of referral vision increased with age from 41.2 to 75.0% across 6- to 12-year age groups (Table 1).

**Table 1:** The prevalence of referral vision and refraction errors, and spherical equivalence refraction before and after cycloplegia among 6- to 12-year-old children in Beijing, No. (%).

	**6 y (570)**	**7 y (1,223)**	**8 y (745)**	**9 y (722)**	**10 y (597)**	**11 y (432)**	**12 y (517)**	**Total (4,806)**	***P*_trend_ value^*a*^**
Referral vision^***b***^, No. (%)	235 (41.2)	451 (36.9)	308 (41.3)	354 (49.0)	367 (61.5)	302 (69.9)	388 (75.0)	2,405 (50.0)	<0.001
**Prevalence of refraction error pre- cycloplegia**									
Myopia, No. (%)	149 (26.1)	436 (35.7)	351 (47.1)	428 (59.3)	414 (69.3)	331 (76.6)	442 (85.5)	2,551 (53.1)	<0.001
Emmetropia, No. (%)	202 (35.4)	467 (38.2)	273 (36.6)	191 (26.5)	127 (21.3)	61 (14.1)	49 (9.5)	1,370 (28.5)	
Mild hyperopia, No. (%)	213 (37.4)	310 (25.3)	113 (15.2)	95 (13.2)	53 (8.9)	34 (7.9)	24 (4.6)	842 (17.5)	
Hyperopia, No. (%)	6 (1.1)	10 (0.8)	8 (1.1)	8 (1.1)	3 (0.5)	6 (1.4)	2 (0.4)	43 (0.9)	
**Prevalence of refraction error post- cycloplegia**									
Myopia, No. (%)	14 (2.5)	120 (9.8)	143 (19.2)	263 (36.4)	308 (51.6)	264 (61.1)	370 (71.6)	1,482 (30.8)	<0.001
Emmetropia, No. (%)	59 (10.4)	146 (11.9)	112 (15.0)	112 (15.5)	89 (14.9)	49 (11.3)	50 (9.7)	617 (12.8)	
Mild hyperopia, No. (%)	446 (78.2)	902 (73.8)	473 (63.5)	335 (46.4)	190 (31.8)	109 (25.2)	91 (17.6)	2,546 (53.0)	
Hyperopia, No. (%)	51 (8.9)	55 (4.5)	17 (2.3)	12 (1.7)	10 (1.7)	10 (2.3)	6 (1.2)	161 (3.3)	
***P*** **-value** ^*c*^	<0.001	<0.001	<0.001	<0.001	<0.001	<0.001	<0.001	<0.001	
SER _pre−cycloplegia_ D, mean (SD)	0.00 (0.93)	−0.19 (1.08)	−0.47 (1.12)	−0.81 (1.28)	−1.19 (1.44)	−1.50 (1.83)	−2.07 (1.88)	−0.75 (1.48)	<0.001
SER_post−cycloplegia_ D, mean (SD)	1.06 (0.81)	0.76 (1.03)	0.38 (1.17)	−0.13 (1.38)	−0.68 (1.62)	−0.99 (1.95)	−1.60 (1.99)	0.01 (1.63)	<0.001
DSER^*d*^, D, mean (SD)	−1.06 (0.97)	−0.95 (0.92)	−0.86 (0.79)	−0.68 (0.70)	−0.51 (0.54)	−0.51 (0.66)	−0.47 (0.89)	−0.76 (0.84)	<0.001
***p*** **-value** ^*e*^	<0.001	<0.001	<0.001	<0.001	<0.001	<0.001	<0.001	<0.001	

a *Ptrend value, trend Chi – square was used to explored the prevalence change trend of refraction errors among 6- to 12- year-old children*.

b *Referral vision, is defined as uncorrected visual acuity lower than 0.0 logMRA, which need to referral to clinic for further examination*.

c *P-value, non-parametric test (k-related samples) to compare the difference of refraction status before and after cycloplegia in the same age children*.

d *DSER, SER pre-cycloplegia – SER post-cycloplegia*.

e *P-value, paired samples T-test to explore the difference between SER pre-cycloplegia and SER post-cycloplegia in the same age children*.

**Table 2:** Composition changes of the refraction status pre- and post-cycloplegia among 6- to 12 years old children in Beijing. No. (%).

**Age y**	**Emmetropia (pre-cycloplegia)**					**Myopia (pre-cycloplegia)**					**Hyperopia (pre-cycloplegia)**				
	***n***	**Emmetropia (post-)**	**Myopia (post-)**	**Hyperopia (post-)**	**p_**trend**_ value**	***n***	**Emmetropia (post-)**	**Myopia (post-)**	**Hyperopia (post-)**	**P_**trend**_ value**	***n***	**Emmetropia (post-)**	**Myopia (post-)**	**Hyperopia (post-)**	**P_**trend**_ value**
6	202	35 (17.3)	1 (0.5)	166 (82.2)	<0.001	149	22 (14.8)	13 (8.7)	114 (76.5)	<0.001	219	2 (0.9)	0 (0.0)	217 (99.1)	0.747
7	467	67 (14.3)	6 (1.3)	394 (84.4)		436	77 (17.7)	113 (25.9)	246 (56.4)		320	2 (0.6)	1 (0.3)	317 (99.1)	
8	273	44 (16.1)	2 (0.7)	227 (83.2)		351	68 (19.4)	141 (40.2)	142 (40.5)		121	0 (0.0)	0 (0.0)	121 (100.0)	
9	191	49 (25.7)	3 (1.6)	139 (72.8)		428	61 (14.3)	260 (60.7)	107 (25.0)		103	2 (1.9)	0 (0.0)	101 (98.1)	
10	127	36 (28.3)	2 (1.6)	89 (70.1)		414	52 (12.6)	305 (73.7)	57 (13.8)		56	1 (1.8)	1 (1.8)	54 (96.4)	
11	61	16 (26.2)	1 (1.6)	44 (72.1)		331	33 (10.0)	263 (79.5)	35 (10.6)		40	0 (0.0)	0 (0.0)	40 (100.0)	
12	49	16 (32.7)	1 (2.0)	32 (65.3)		442	34 (7.7)	369 (83.5)	39 (8.8)		26	0 (0.0)	0 (0.0)	26 (100.0)	
Total	1,370	263 (19.2)	16 (1.2)	1,091 (79.6)		2,551	347 (13.6)	1,464 (57.4)	740 (29.0)		885	7 (0.8)	2 (0.2)	876 (99.0)	

**Figure 1:**
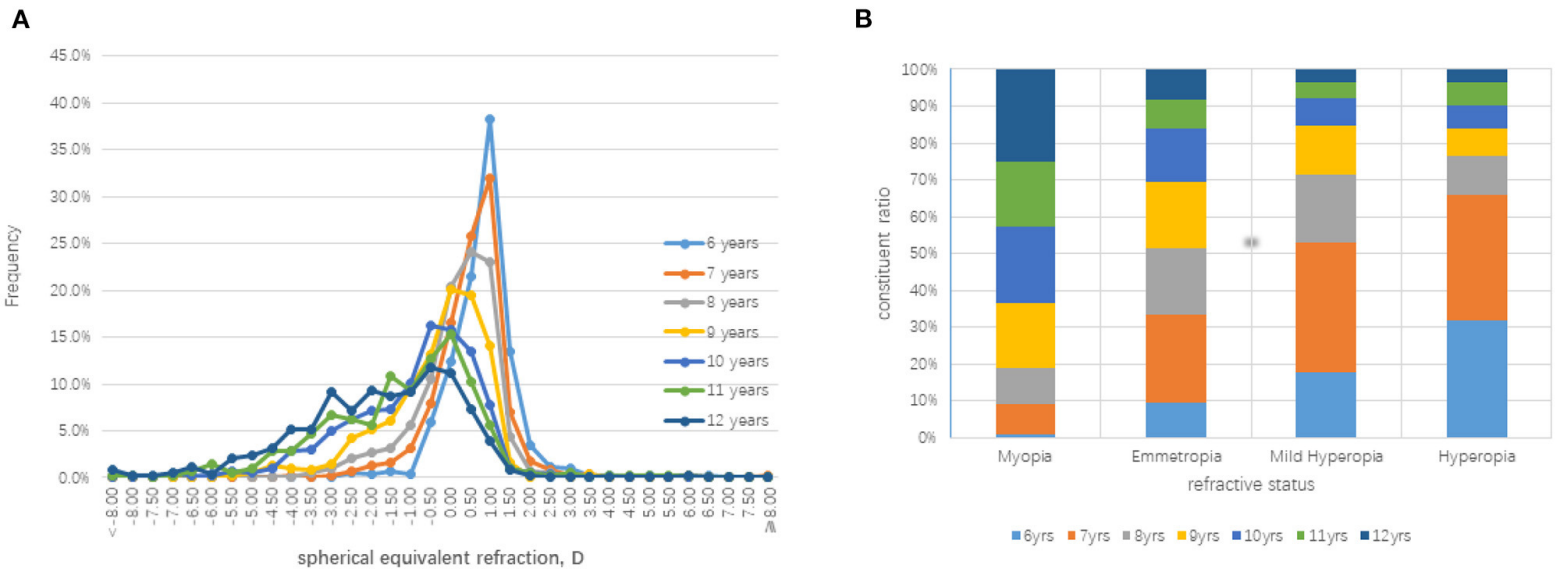
Distributions of the cycloplegia spherical equivalent refraction (SER) in the right eye of the each age group. **(A)** The frequency distributions of SER among different age children. **(B)** The composition ratio of different ages children among the different refractive error groups.

Table 1 and Figure 1 show the distribution of SER and demonstrate myopic shift as the students' ages increased. There was a significant difference between the non-cycloplegia and cycloplegia refractions, which was −0.76±0.84 D. The average SER difference between the same age group decreased from −1.06 D at 6- to −0.47 D at 12- years old as their age increased. A total of 19.2, 57.4, and 99.0% of children in the emmetropia, myopia and hyperopia groups, respectively, had consistent results between pre- and post-cycloplegia (Table 2). Among the students with myopia before cycloplegia, with increasing age, the prevalence of diagnosed myopia after cycloplegia was increased. The proportion significantly increased from 8.7% in the 6- to 83.5% in the 12-year-old group. Among all the students classified as emmetropia with non-cycloplegia, 79.6% of them became hyperopia after cycloplegia.

The averaged parameters of the ocular biometry by age are summarized in Table 3. The AL increased with age across all of the refractive error groups (F_trend_ = 2002.431, ρ_trend_ <0.001), but there were variations in the growth rate among the different refraction statuses and different age groups. Overall, the AL elongation grew the fastest from 8 to 9 years old, and it increased by approximately 0.41 mm/year. In the emmetropia and hyperopia groups, the annual increase was similar and was approximately 0.15 mm/year every addition year, while in the myopia group, it was 0.19 mm/year. In addition, the slowest growth rate appeared in the hyperopia group (Table 3 and Supplementary Figure 2).

**Table 3:** Distribution of the biometry parameters among different refraction error groups in 6- to 12 years old children, Beijing.

**Parameters**	**6 y**	**7 y**	**8 y**	**9 y**	**10 y**	**11 y**	**12 y**	**Total**	***p*_trend_ value^*a*^**
**Axial length, mm, mean (SD)**									
Myopia	23.46 (0.90)	23.50 (0.70)	23.74 (0.74)	23.96 (0.82)	24.21 (0.86)	24.41 (0.94)	24.60 (0.97)	24.19 (0.94)	<0.001
Emmetropia	22.86 (0.55)	23.06 (0.60)	23.20 (0.63)	23.49 (0.65)	23.33 (0.75)	23.54 (0.59)	23.75 (0.93)	23.28 (0.71)	<0.001
Hyperopia	22.39 (0.68)	22.63 (0.66)	22.80 (0.67)	23.05 (0.710	23.08 (0.74)	23.10 (0.79)	23.26 (0.71)	22.75 (0.73)	<0.001
Total	22.46 (0.70)	22.77 (0.71)	23.04 (0.77)	23.45 (0.85)	23.70 (0.97)	23.95 (1.05)	24.26 (1.07)	23.26 (1.03)	<0.001
**p**_**trend**_ **value**^*b*^	<0.001	<0.001	<0.001	<0.001	<0.001	<0.001	<0.001	0.001	
**AL-to-CR**									
Myopia	3.01 (0.08)	3.04 (0.08)	3.08 (0.08)	3.09 (0.08)	3.13 (0.09)	3.14 (0.11)	3.17 (0.11)	3.12 (0.10)	<0.001
Emmetropia	2.97 (0.05)	2.98 (0.05)	3.01 (0.05)	3.01 (0.05)	3.01 (0.06)	3.00 (0.12)	3.03 (0.09)	3.00 (0.06)	<0.001
Hyperopia	2.90 (0.08)	2.92 (0.06)	2.94 (0.06)	2.96 (0.06)	2.96 (0.07)	2.96 (0.08)	2.98 (0.07)	2.93 (0.07)	<0.001
Total	2.91 (0.08)	2.94 (0.07)	2.98 (0.09)	3.01 (0.09)	3.06 (0.11)	3.07 (0.13)	3.12 (0.13)	3.00 (0.12)	<0.001
**p**_**trend**_ **value**^*b*^	<0.001	<0.001	<0.001	<0.001	0.016	0.042	<0.001	<0.001	
**Corneal power, D, mean (SD)**									
Myopia	43.37 (1.72)	43.76 (1.55)	43.88 (1.32)	43.50 (1.47)	43.65 (1.33)	43.42 (1.61)	43.47 (1.35)	43.57 (1.44)	0.624
Emmetropia	43.80 (1.18)	43.58 (1.43)	43.76 (1.38)	43.23 (1.33)	43.63 (1.54)	43.05 (1.94)	43.09 (1.75)	43.49 (1.49)	0.001
Hyperopia	43.72 (1.68)	43.53 (1.42)	43.50 (1.39)	43.3891.43)	43.36 (1.52)	43.34 (1.41)	43.24 (1.33)	43.50 (1.48)	0.001
Total	43.72 (1.64)	43.56 (1.43)	43.61 (1.38)	43.40 (1.43)	43.55 (1.43)	43.36 (1.60)	43.39 (1.39)	43.52 (1.47)	<0.001
***p*_trend_ **value**^*b*^**	0.727	0.601	0.073	0.350	0.146	0.275	0.545	0.856	
**Anterior chamber depth, mm, mean (SD)**									
Myopia	3.46 (0.31)	3.59 (0.25)	3.62 (0.26)	3.63 (0.25)	3.71 (0.28)	3.73 (0.27)	3.76 (0.27)	3.69 (0.27)	<0.001
Emmetropia	3.47 (0.24)	3.51 (0.24)	3.53 (0.22)	3.55 (0.30)	3.58 (0.25)	3.54 (0.40)	3.64 (0.30)	3.53 (0.27)	0.02
Hyperopia	3.37 (0.28)	3.37 (0.25)	3.42 (0.25)	3.48 (0.27)	3.49 (0.24)	3.48 (0.35)	3.49 (0.31)	3.41 (0.27)	<0.001
Total	3.38 (0.28)	3.41 (0.26)	3.47 (0.26)	3.54 (0.28)	3.62 (0.28)	3.64 (0.33)	3.70 (0.30)	3,51 (0.30)	<0.001
**p**_**trend**_ **value**^*b*^	0.012	<0.001	<0.001	0.019	0.005	0.213	0.002	<0.001	
**Lens power, D, mean (SD)**									
Myopia	24.80 (1.62)	24.08 (1.44)	23.28 (1.48)	23.14 (1.40)	22.53 (1.45)	22.41 (1.99)	22.19 (1.61)	22.75 (1.70)	<0.001
Emmetropia	24.59 (1.51)	24.21 (1.23)	23.41 (1.24)	23.00 (1.54)	23.27 (1.45)	23.06 (2.03)	22.44 (2.66)	23.51 (1.69)	<0.001
Hyperopia	25.06 (1.88)	24.41 (1.38)	23.94 (1.39)	23.25 (1.52)	23.29 (1.37)	22.93 (1.62)	22.73 (1.53)	24.09 (1.67)	<0.001
Total	25.01 (1.84)	24.35 (1.37)	23.74 (1.42)	23.17 (1.48)	22.90 (1.47)	22.63 (1.92)	22.31 (1.74)	23.60 (1.78)	<0.001
**p**_**trend**_ **value**^*b*^	0.067	0.107	<0.001	0.125	0.930	0674	0.341	<0.001	
**Corneal radius, mm, mean (SD)**									
Myopia	7.79 (0.32)	7.72 (0.27)	7.70 (0.23)	7.77 (0.26)	7.74 (0.23)	7.78 (0.33)	7.75 (0.46)	7.75 (0.33)	0.898
Emmetropia	7.71 (0.21)	7.75 (0.25)	7.72 (0.24)	7.81 (0.24)	7.75 (0.27)	7.86 (0.42)	7.84 (0.32)	7.77 (0.27)	0.001
Hyperopia	7.73 (0.39)	7.76 (0.25)	7.77 (0.25)	7.79 (0.26)	7.79 (0.28)	7.79 (0.26)	7.81 (0.24)	7.77 (0.29)	0.004
Total	7.73 (0.38)	7.76 (0.25)	7.75 (0.25)	7.79 (0.26)	7.76 (0.26)	7.80 (0.32)	7.77 (0.42)	7.76 (0.30)	0.002
**p**_**trend**_ **value**^*b*^	0.661	0.663	0.072	0.367	0.146	0.243	0.659	0.881	

a *P_trend_ value, AONVA was used to explored the change trend of biometry parameters among 6- to 12- year-old children*.

b *P_trend_ value, AONVA was used to compare the change trend of biometry parameters in different refraction status in the same age children*.

The ACD was increased from 3.38 ± 0.28 mm to 3.70 ± 0.30 mm from 6 to 12 years of age. The calculated Bennett-Rabbetts LP showed a declining trend as age increased, with an average of 25.01 ± 1.84 D at 6- and 22.31 ± 1.74 at 12- years of age. In addition, the AL-to-CR ratio increased with age, with a mean of 2.91 ± 0.08 in the 6- and increased to 3.12 ± 0.13 in the 12-year-old group. The change in the SER with the AL-to-CR ratio was linear, and on average, 1-unit difference in the AL-to-CR ratio was associated with a −0.82 D difference in the SER. It shows prominent increased amplitude of the AL-to-CR ratio in myopia group than the other groups (Table 3 and Supplementary Figure 3).

Multiple linear regression models were fitted to explore the associations between the SER and ocular biometry after adjusting for age and gender (Table 4). When only the AL was included in the model, 56.4% of the variance in SER could be explained, with a 1-mm increase in AL associated with −0.65 D SER. Model 3 assessed the association between the SER and the AL-to-CR ratio, which explained 72.4% of the variance. An increase of 1 unit in the AL-to-CR ratio was associated with a −0.82D change in the SER. Comparing to Model 2 and Model 4, the CP explained better than CR, and inversely associated with the SER. Model 5 included the calculated LP, AL, and CR, which explained 86.4% of the variance in the SER.

**Table 4:** Linear regression models for SER(OD)-C with age, gender, and ocular biometry.

**Variables**	**Model 1**		**Model 2**		**Model 3**		**Model 4**		**Model 5**	
	**β**	***P***	**β**	***P***	**β**	***P***	**β**	***P***	**β**	***P***
Age	−0.159	<0.001	−0.032	0.001	−0.054	<0.001	0.024	0.006	−0.069	<0.001
Gender^*a*^	0.036	<0.001	0.043	<0.001	0.024	0.002	0.042	<0.001	0.015	0.004
AL (OD, mm)	−0.653	<0.001	−0.915	0.001			−1.045	<0.001	−1.352	<0.001
CR (OD, mm)			0.436	<0.001					0.607	<0.001
CP (OD, D)							−0.561	<0.001		
LP (OD, D)									−0.578	<0.001
AL-to-CR (OD)					−0.820	<0.001				
*R* ^2^	0.564		0.706		0.724		0.771		0.864	

a *Adjusted for age and sex (girls as reference)*.

## Discussion

The present BPCE study aims to investigate the baseline characteristics of the refraction and ocular biometry parameters in primary school students aged between 6 and 12 years after COVID-19. Our findings included: (1) The prevalence of emmetropia and myopia was 12.8 and 30.8% under cycloplegia, respectively, which was lower than that of non-cycloplegia conditions; (2) The myopia prevalence increased significantly, with an ascending trend increasing from 2.5% at 6- to 71.6% at 12 years old, with the speed increase 27.6%. And the 9- and 10-year ages increased most prominently; (3) The median cycloplegic SER was significantly higher than non-cycloplegia SER. A total of 57.4% of the myopic students before cycloplegia remain diagnosed as myopia after cycloplegia; (4) Along with the AL, CR and LP after adjusting age and gender, the computed model explained 86.4% of the variance in the SER.

### The Prevalence of Myopia Increased Dramatically With Age

There are variations in the prevalence of myopia between different regions and ethnic groups (15, 16). As reported by Zhou and colleagues, it has been universally accepted that Chinese schoolchildren have a higher prevalence of myopia at a given age in population-based epidemiology study, and highly related to less time spend out doors and intense education (17). However, in view of the current situation in exposure to electronic screens, intensive early education, and especially the lockdowns caused by COVID-19, the distribution of emmetropia, ametropia and the prevalence of myopia need to be investigated further. Pang P and colleagues reported (18) that the overall myopia prevalence under non-cycloplegia refraction after COVID-19 was 42.82, 75.25, and 82.57% in elementary school, junior high school and senior high school, respectively. Restricted outdoor time and increased screen time are contributors to increased myopia during quarantine.

Evidence from the present BPCE study showed a substantially higher prevalence of myopia, increasing by 10–15% across all age groups comparing to recently reported results, (6, 19–21) and this possibly reflects the trends for the earlier development of myopia in Beijing Pinggu District children. Particularly surprising is that, in 9- and 10-year-olds showing the most prominent increases. This may be due to the characteristics of Chinese education, which in Grade 3 to Grade 5, the schoolwork pressure suddenly increased, and students began to endure intense out-of-class activities, such as English and Mathematical Olympiad, etc. However, as reported by several studies, the non-cycloplegic refraction is inaccurate for studying the refractive error in children, especially those under 12 years of age (22, 23). In the present study, a greater discrepancy between the cycloplegia and non-cycloplegia autorefraction results was found. As shown in Table 1, the detection rate of myopia and emmetropia under cycloplegic treatment decreased by 22.3 percent points and 15.7 percent points compared with that before cycloplegia, respectively, while the prevalence of hyperopia increased. After cycloplegia, 42.6% of myopic children became emmetropia or hyperopia. Cycloplegia have a greater effects for younger children than those older children. Among 6 years old children, 91.3% of myopic children became emmetropia or hyperopia, but only 26.5% among those in 12 years old remained myopic. So the prevalence of myopia before and after cycloplegia became gradually closer to each other as age increased. In Anyang study, it was also suggested Chinese children with myopia may not be accurately diagnosed. So the prevalence of myopia before and after cycloplegia became gradually closer to each other as age increased.

### The Refraction Status Distribution Varied Significantly Between Pre- and Post-cycloplegia

It is widely acknowledged that differences between non-cycloplegic and cycloplegic refractions were more marked at younger ages (22–25). The non-cycloplegic condition includes a strong accommodative component, especially in younger children with dark irises. Inadequate cycloplegia can lead to a marked increase in the prevalence of myopia that is reported. In the present study, compared to cycloplegia data, the non-cycloplegic SER was −0.76 D (range from −1.06 D to −0.47 D), which is more myopic than the cycloplegic refraction. The prevalence of myopia of non-cycloplegis is significantly increased compared to non-cycloplegia SER, and the difference ranges from 23.6% at 6 years old to 13.9% at 12 years old. With this strict condition of cycloplegic eye drop administration, it is concluded that the prevalence in primary school children is substantially increased (11) and is higher than that in other cities in China, such as in Guangzhou, compared to the same age group (26, 27).

The present finding is consistent with Liu et al.'s (27) report that without cycloplegia, autorefraction tends to overestimate the SER in children with myopia and is negatively correlated with age. For an accurate diagnosis and effective myopia management strategies, cycloplegia should be used for children in primary school students between 6 and 12 years of age according to the present study.

### Ocular Biometry Factors Distribution and Relationship to the Myopic Status

The eye grows rapidly during early childhood and then grows slowly during the later stages (28). It has been reported that each 0.1 mm in the eye elongation corresponds to an approximately 0.20–0.25 D myopic change (29, 30). Consistent with previously reported articles, the AL increased with age across all of the refractive error groups, but there were variations among the different refraction statuses and different age groups. For the emmetropia and hyperopia groups, the average eye growth rate was 0.15 mm/year from 6 to 12 years of age, while the rate was approximately 0.19 mm/year in the myopia group, which was faster than that in non-myopic students. It is more interesting that, if the 6-year-old group was excluded, and if only the age 7- to 12-year-old group of students were analyzed, the eye growth rate in myopic children was ~0.22 mm/year, which is faster than normal emmetropazation condition, indicating the shift to myopia development.

We found the same results as previously reported: the ACD deepened and the lens power decreased with increasing age among 6- to 12-year-old children. The AL-to-CR ratio is supposed to be a synthetic indicator to determine the refractive state of the human eye, and no <3.0 has been considered as an indicator for the onset of myopia (13). Our present study results showed that a higher AL-to-CR ratio correlated with myopia prevalence and a negative refractive error status, which explained 72.4% of the SER variance. In the present study, the average AL-to-CR ratio was 3.12 in myopia children, and the increment was 0.16 from 6 to 12 years old in myopia group. However, the increase of the AL-to-CR ratios in emmetropia group and hyperopia group were 0.06 and 0.08, respectively. In addition, when including AL, CR and LP in prediction computation model, they can explain 86.4% of the variance in the SER, in which LP and AL was found to be inversely associated with the SER.

### Limitations

There were several limitations in the present study, including that there was no direct measurement data of the lens thickness and lens power, although they have been calculated through validated forums. In addition, there were no records about the parental history, duration of near work, screen use, or outdoor activity time. The above information will be collected through questionnaires during subsequent screening studies. Despite the limitations, the relative changes imply that these are valuable indicators for evaluating refraction and ocular biometry parameters.

## Conclusions

In this study, the prevalence of myopia was increased compared to previous studies in primary school-age children. More importantly, the results indicate the significance of cycloplegia refraction is a more accurate method for epidemic investigation. The current myopia screening strategy is mainly based on non-mydriatic SER results, although high-risk groups can be detected in early stage, it may lead to false positives phenomenon, especially in younger students' group. These findings contribute further insights into the importance of disseminating the notion of myopia intervention strategies and regular follow-up for school-age students in China. Long-term investigations are needed to determine further changes in the refraction status in China.

## Data Availability Statement

The raw data supporting the conclusions of this article will be made available by the authors, without undue reservation.

## Ethics Statement

The studies involving human participants were reviewed and approved by ethics approval was obtained from the Institutional Review Board of the Peking University People's Hospital. Written informed consent to participate in this study was provided by the participants' legal guardian/next of kin.

## Author Contributions

YL, KW, MZ, and JQ initiated the study design. JM, JZ, CJ, and CZ prepared the consent form. HZ, LW, WW, XL, and XG drafted and finalized the study protocol. YL and YX wrote the draft. All authors reviewed the study protocol and approved the final manuscript.

## Funding

This work was supported by National Natural Science Foundation of China (Grant Nos. 82171092 and 81870684), National Key R&D Program of China (Nos. 2020YFC2008200 and 2021YFC2702100), Peking University People's Hospital Scientific Research Development Funds (RDH2021-03), and Capital's Funds for Health Improvement and Research (No. 2022-1G-4083).

## Conflict of Interest

The authors declare that the research was conducted in the absence of any commercial or financial relationships that could be construed as a potential conflict of interest. The handling editor YZ declared a shared parent affiliation with the authors YL, YX, JM, XL, JZ, CZ, MZ, and KW at the time of review.

## Publisher's Note

All claims expressed in this article are solely those of the authors and do not necessarily represent those of their affiliated organizations, or those of the publisher, the editors and the reviewers. Any product that may be evaluated in this article, or claim that may be made by its manufacturer, is not guaranteed or endorsed by the publisher.
