# Supplementary figures and images for: Beijing Pinggu Childhood Eye Study: The Baseline Refractive Characteristics in 6- to 12-Year-Old Chinese Primary School Students
Source: Front Public Health. 2022 May 27;10:890261. doi: 10.3389/fpubh.2022.890261 (PMC9196872; doi:10.3389/fpubh.2022.890261)

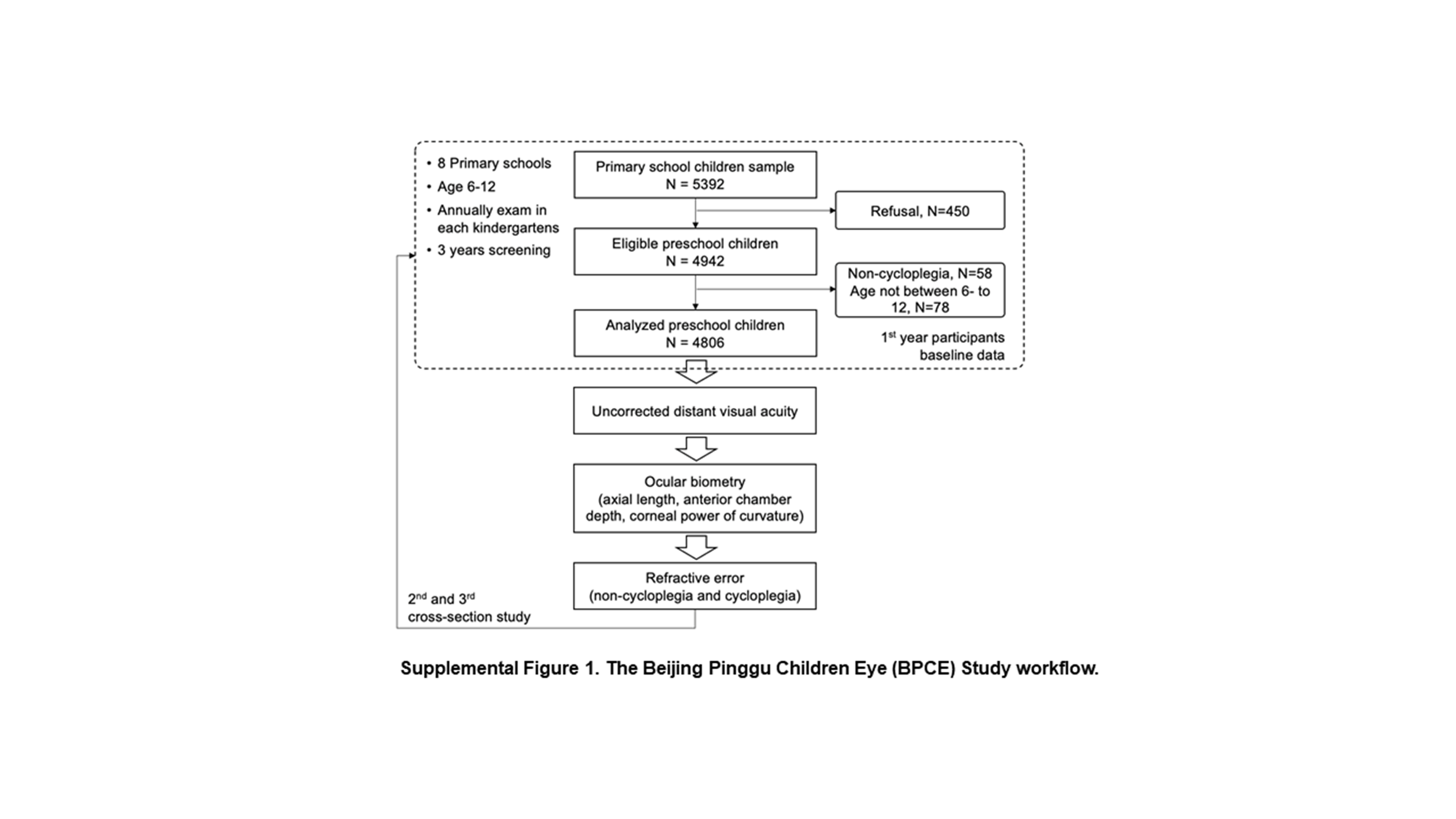

Supplement: Supplementary file 1 [file Image_1.tif]

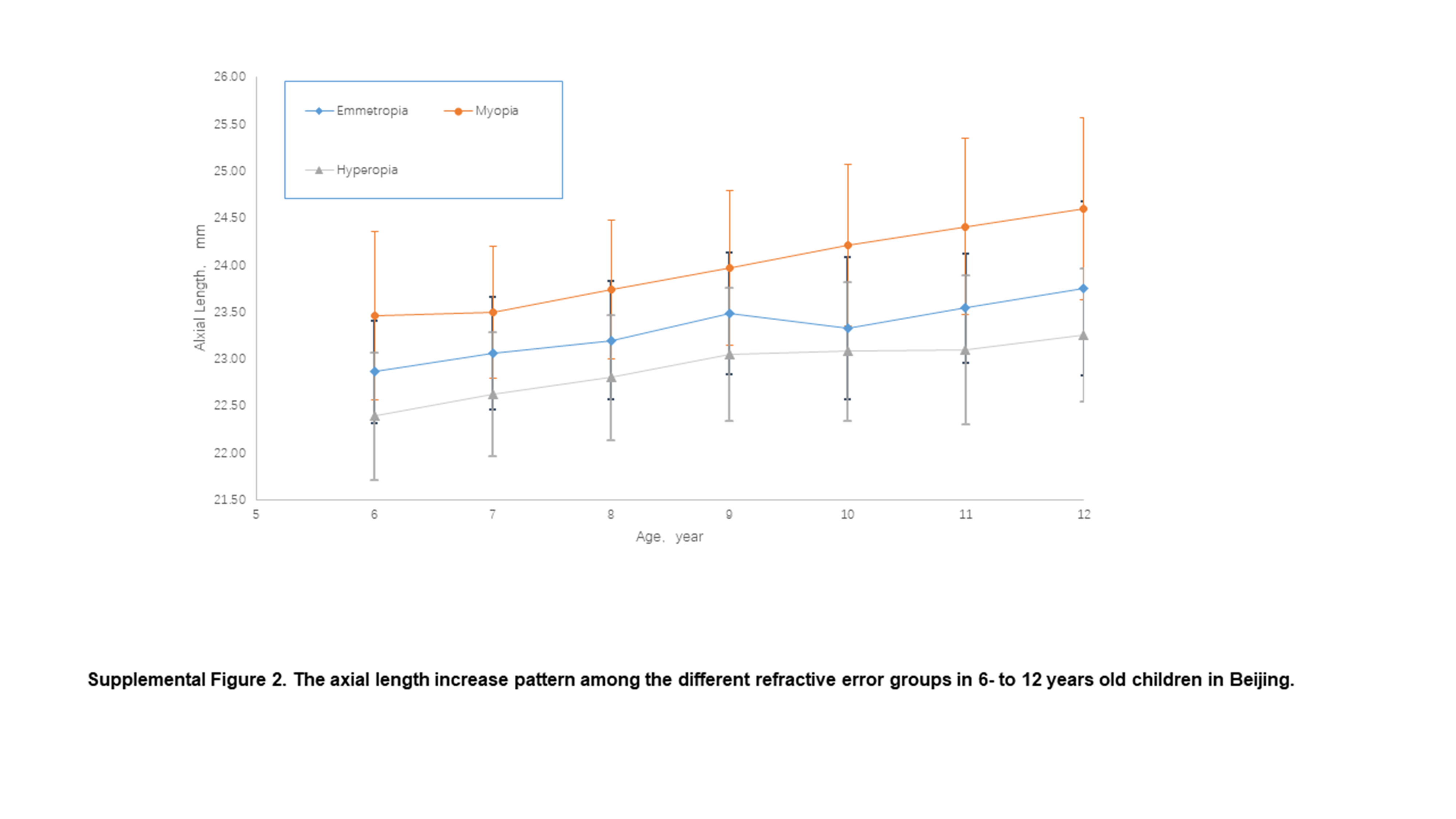

Supplement: Supplementary file 2 [file Image_2.tif]

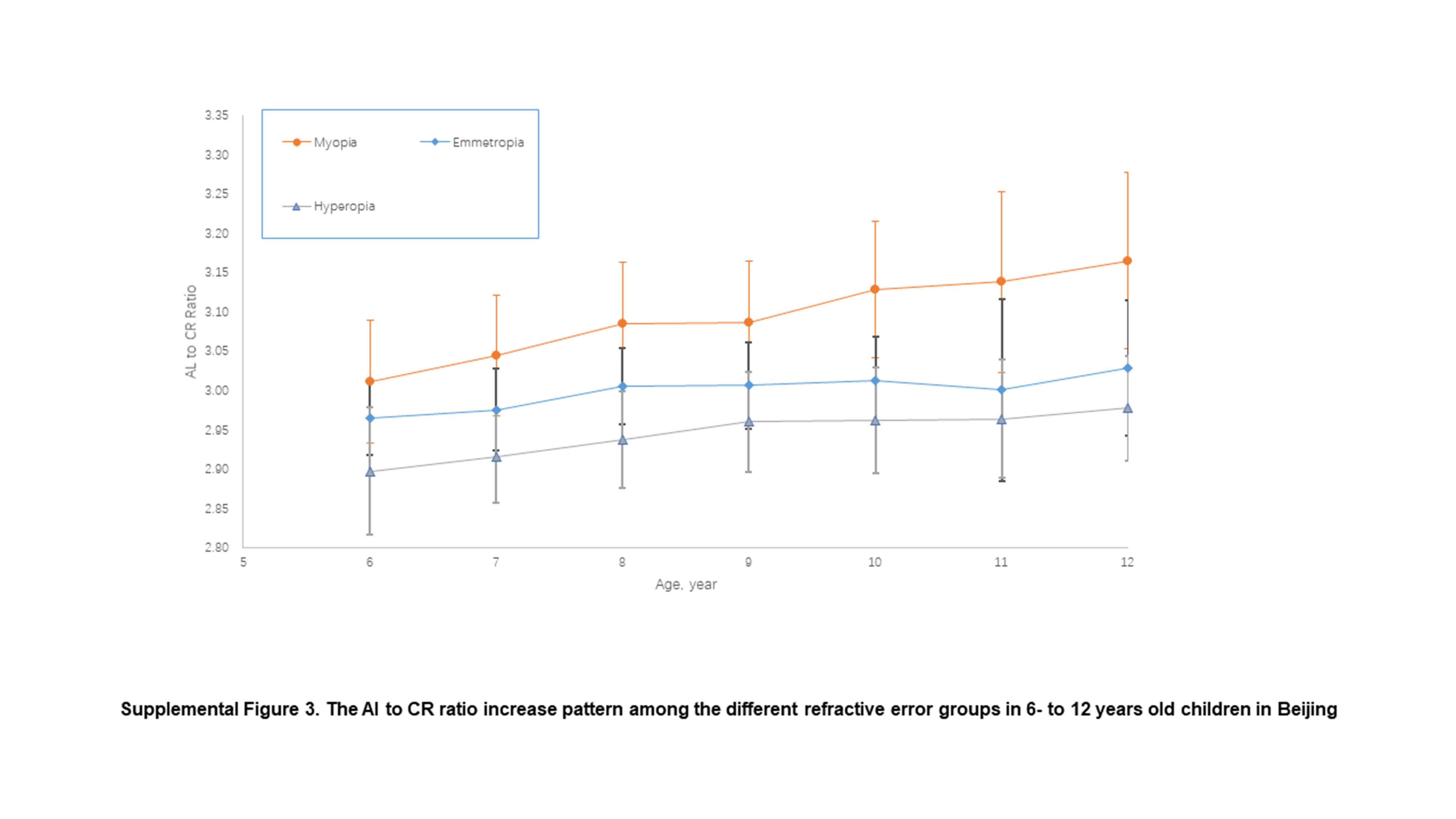

Supplement: Supplementary file 3 [file Image_3.tif]
